# Med1 controls CD8 T cell maintenance through IL‐7R‐mediated cell survival signalling

**DOI:** 10.1111/jcmm.16465

**Published:** 2021-03-17

**Authors:** Lei Lei, Xiaofeng Yang, Yanhong Su, Huiqiang Zheng, Jun Liu, Haiyan Liu, Yujing Zou, Anjun Jiao, Xin Wang, Cangang Zhang, Xingzhe Zhang, Jiahui Zhang, Dan Zhang, Xiaobo Zhou, Lin Shi, Enqi Liu, Liang Bai, Chenming Sun, Baojun Zhang

**Affiliations:** ^1^ Department of Pathogenic Microbiology and Immunology School of Basic Medical Sciences Xi’an Jiaotong University Xi’an China; ^2^ Institute of Infection and Immunity Translational Medicine Institute Xi’an Jiaotong University Health Science Center Xi’an China; ^3^ Key Laboratory of Environment and Genes Related to Diseases (Xi'an Jiaotong University) Ministry of Education Xi'an China; ^4^ Xi’an Key Laboratory of Immune Related Diseases Xi’an China; ^5^ Duke University Medical Center Durham NC USA; ^6^ Institute of Cardiovascular Science Xi'an Jiaotong University Health Science Center Xi'an China

**Keywords:** apoptosis, CD8^+^ T cells, homeostasis, IL‐7R signalling, Med1

## Abstract

Under steady‐state conditions, the pool size of peripheral CD8^+^ T cells is maintained through turnover and survival. Beyond TCR and IL‐7R signals, the underlying mechanisms are less well understood. In the present study, we found a significant reduction of CD8^+^ T cell proportion in spleens but not in thymi of mice with T cell‐specific deletion of Mediator Subunit 1 (Med1). A competitive transfer of wild‐type (WT) and Med1‐deficient CD8^+^ T cells reproduced the phenotype in the same recipients and confirmed intrinsic role of Med1. Furthermore, we observed a comparable degree of migration and proliferation but a significant increase of cell death in Med1‐deficient CD8^+^ T cells compared with WT counterparts. Finally, Med1‐deficient CD8^+^ T cells exhibited a decreased expression of interleukin‐7 receptor α (IL‐7Rα), down‐regulation of phosphorylated‐STAT5 (pSTAT5) and Bim up‐regulation. Collectively, our study reveals a novel role of Med1 in the maintenance of CD8^+^ T cells through IL‐7Rα/STAT5 pathway‐mediated cell survival.

## 1 INTRODUCTION

CD8^+^ T cells, also known as cytotoxic T cells, are pivotal for the immune defence against pathogen infection and tumour growth.^1^ The pool size and status of peripheral CD8^+^ T cells determine the power of immune response. Basal TCR signal induced by contact with self‐peptide/MHC ligands is necessary for naïve T cell survival and homeostatic proliferation, as deprivation of MHC I molecules or ablating TCR expression renders naïve T cells sensitive to cell death.^2^ Basal TCR signalling induces pro‐survival molecules expression indirectly by augmenting responsiveness to cytokines.^3^ Cytokine signals are also responsible for naïve T cell maintenance in the periphery by controlling homeostatic proliferation and survival.^4^, ^5^

Among well‐known cytokines, Interleukin‐7 (IL‐7) is a key cytokine required for the survival and proliferation of peripheral naïve CD8^+^ T cells.^6^, ^7^, ^8^ IL‐7Rα forms a heterodimer with common γc chain.^9^ The surface domain binds to IL‐7 cytokine, while the intercellular domain bridges JAK‐1 and JAK‐3. Two JAK proteins phosphorylate each other and activate STAT5 downstream, as well as PI3K‐Akt‐mTOR and MEK‐ERK pathways.^10^ IL‐7/IL‐7Rα‐mediated signals maintain T cell survival by inducing the expression of pro‐survival factors Bcl2 and Mcl1, and down‐regulating proapoptotic proteins Bad, Bax, Bim etc.^11^, ^12^, ^13^, ^14^, ^15^

Mediator, as a large complex composed of 25‐30 subunits, which is a highly conserved and integral part of RNAP II‐mediated transcriptional machinery of the eukaryotes. It serves as a molecular bridge between transcription factors and the RNA polymerase II.^16^ Med1, as a key component of mediator complex, is associated with the binding of a variety of cofactors to regulate gene transcription.^17^ Med1 can promote cell proliferation and survival through interacting with transcription factors, such as p53,^18^ GATA family proteins^19^, ^20^ and PPARα,^21^ and contribute to the pathogenesis of various of cancer, including breast cancer, hepatoma and prostate cancer.^22^, ^23^, ^24^ Med1 mutation in mice results in embryonic and early postnatal lethality.^25^ It was recently reported that Med1 deletion could impair the transition of developmental stages in thymic invariant natural killer T (iNKT) cells without affecting thymic αβ T cells.^26^ A reduction of CD8^+^ T cell population in the spleen was also mentioned. However, it is unclear whether Med1 plays an intrinsic role and what is the involved mechanism.

In the present study, we report that Med1 deletion in T cells intrinsically impaired CD8^+^ T cell population size in the periphery of unchallenged mice. Med1‐deficient CD8^+^ T cells exhibited an increase in apoptosis. Mechanistically, Med1 deletion caused a reduction in IL‐7Rα expression, reduction of pSTAT5 expression downstream as well as up‐regulation of Bim. In summary, our study reveals Med1 is required for CD8^+^ T cell survival and homeostatic maintenance.

## 2 MATERIALS AND METHODS

### 2.1 Mice

The Med1^f/f^ strain was generated as previously described.^27^ Med1^TKO^ mice (LckCre^+^Med1^f/f^) were generated by crossing Med1^f/f^ mice with LckCre transgenic strain. All mice were bred and maintained in the specific pathogen‐free conditions by Xi'an Jiaotong University Division of Laboratory Animal Research. All the procedures were approved by the Institutional Animal Care and Use Committee of Xi'an Jiaotong University.

### 2.2 Antibodies and reagents

The Abs used are as follows: APC/Cy7 anti‐mouse CD4 (clone GK1.5), FITC anti‐mouse CD8a (clone 53‐6.7), APC/Cy7 anti‐mouse CD8 (clone 53‐6.7), PE/Cy7 anti‐mouse/human CD44 (clone IM7), APC anti‐mouse CD62L (clone MEL‐14), FITC anti‐BrdU (clone 3D4), PE anti‐Annexin V (Cat # 640947), PE/Cy7 anti‐mouse CD127(IL‐7R) (clone A7R34), APC/Cy7 anti‐mouse CD45.1 (clone A20), FITC anti‐mouse CD45.2 (clone 104), Fixation Buffer(Cat # 420801) and Intracellular Staining Perm Wash Buffer(Cat # 421002). All reagents were purchased from BioLegend. PE anti‐Mo/Rt Ki‐67, (clone SolA15), Transcription Factor Fixation/Permeabilization Concentrate and Diluent were purchased from eBioscience. The APC BrdU Flow Kit was purchased from BD Biosciences. Quick‐RNA Microprep Kit (Cat # R1051) was obtained from Zymo Research.

### 2.3 Flow cytometry and sorting

Single cells were obtained from thymi and spleens of indicated mice. For cell surface analysis, a total of 1 ~ 5 × 10^6^ cells were stained with indicated Abs in the dark at 4°C for 30 minutes. After washing with cold FACS buffer (1× PBS supplemented with 2% FBS), cells were analysed using CytoFLEX flow cytometer (Beckman Coulter). Flowjo software (CytExpert) was used for data analysis. Naïve CD8^+^ T cells (CD8^+^CD44^‐^CD62L^+^) were sorted by BD FACSAria™ Ⅱ cell sorter (BD Biosciences).

### 2.4 T cell culture

Purified naïve CD8^+^ T cells were cultured in RPMI 1640 medium (GIBCO) supplemented with 100 U/mL of penicillin, 100 μg/mL of streptomycin, 0.05 mmol/L of β‐mercaptoethanol and 10% foetal bovin serum (GIBCO) with 10 ng/mL IL‐7 for 5 days. Cells were stained with indicated abs and analysed by flow cytometry.

### 2.5 Bone marrow chimaera

Bone marrow cells from spleens of WT (CD45.2^+^) and Med1^TKO^ mice (CD45.1^+^) were isolated and stained with lineage antibodies (anti‐CD4, CD8, CD3, CD19, CD11b, CD11c and TER‐119). The progenitor cells (lineage negative) were sorted by flow cytometry. Donor cells were mixed at a 1:1 ratio and adoptively transferred intravenously into age‐ and sex‐matched, lethally irradiated (7.5 Gy) WT mice (CD45.1^+^CD45.2^+^). The thymocytes and splenocytes were analysed by flow cytometry 7 weeks after transfer.

### 2.6 T cell adoptive transfer

Naïve CD8^+^ T cells were purified from spleens of WT (CD45.1^+^) and Med1^TKO^ mice (CD45.2^+^), mixed at a 1:1 ratio, and adoptively transferred intravenously into age‐ and sex‐matched, sublethally irradiated (2 Gy) WT mice (CD45.1^+^CD45.2^+^). The splenocytes were collected 24, 72 hours and 1 week after transfer and analysed by flow cytometry.

### 2.7 Quantitative PCR

Quick‐RNA Microprep Kit (Zymo Research) was used to extract total RNA from purified CD8^+^ T cells following the manufacturer's instruction. cDNA was synthesized by the cDNA synthesis kit (TOYOBO). Quantitative PCR was performed on StepOnePlus™ Real‐Time PCR System (ABI) using SYBR Green RT‐qPCR Mastermix (GenStar).

### 2.8 Western blotting

Purified naïve CD8+ T cells were lysed in RIPA lysis buffer (Beyotime Biotechnology) to extract the total proteins. Samples were separated on SDS‐polyacrylamide gels and electro‐transferred onto Polyvinylidene fluoride (PVDF) membranes (Millipore). After blocking with 5% skimmed milk, the membranes were incubated with indicated Med1, STAT5, pSTAT5 and Bim antibodies at 4°C overnight. The membranes were then incubated with secondary antibodies at room temperature for 1h. The protein expression was measured by Fusion‐Solo.6s (VILBER).

### 2.9 Statistics

Data are presented as mean ± SEM. Statistical significance was analysed using the GraphPad Prism 7.0 statistical program. All comparisons between two experimental groups were performed with an unpaired two‐tailed Student's *t* test. The levels of significance are indicated as follows: *P < .05, **P < .01 and ***P < .001.

## 3 RESULTS

### 3.1 A significant reduction of CD8^+^ T cells in the spleen but not in the thymus of Med1‐deficient mice

**FIGURE 1:**
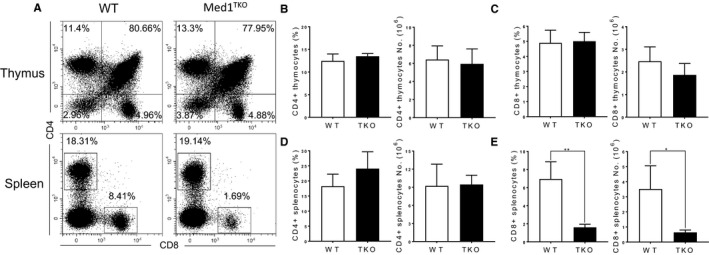
The percentage and number of CD4^+^ and CD8^+^ T cells in the thymus and spleen of WT and Med1^TKO^ mice. Thymocytes and splenocytes from 8‐week‐old WT and Med1^TKO^ mice were stained with anti‐CD4 and anti‐CD8 antibodies. A, Representative FACS plots of CD4 and CD8 expression in thymocytes and splenocytes. B, The percentage and number of CD4^+^ thymocytes. C, The percentage and number of CD8^+^ thymocytes. B, The percentage and number of CD4^+^ splenocytes. C, The percentage and number of CD8^+^ splenocytes. A‐D, Results shown are from one experiment representative of three independent experiments with a total of nine mice per group. *P < .05 and **P < .01 was considered statistically significant and extremely significant, respectively

To study whether Med1 regulates T cell development, we generated mice with specific deletion of Med1 in T cells (Lckcre^+^Med1^f/f^, Med1^TKO^) by crossing Med1^flox/flox^ mice with Lck transgenic strain. We found similar percentages and numbers of CD4^+^ and CD8^+^ T cells in the thymi of WT and Med1^TKO^ groups (Figure 1A‐C). Interestingly, Med1^TKO^ mice exhibited a ~fivefold reduction in CD8^+^ T cell percentage and numbers (Figure 1A,E), but similar proportion of CD4^+^ T cells in spleen compared with WT mice (Figure 1D). Therefore, loss of Med1 impaired CD8^+^ T cell population in the periphery without affecting early T cell development in the thymus.

### 3.2 An intrinsic role of Med1 in peripheral CD8^+^ T cell population

**FIGURE 2:**
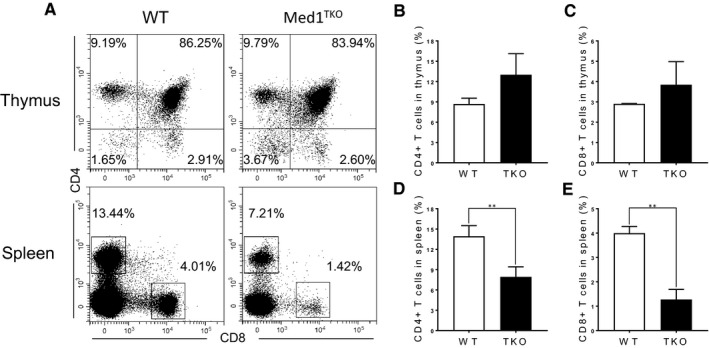
Med1 is intrinsically required for peripheral CD8^+^ T cell population maintenance. The chimeric mice were generated by transplanting a mixed population of WT (CD45.2^+^) and Med1^TKO^ (CD45.1^+^) bone marrow progenitor cells at a 1:1 ratio into lethally irradiated (7.5 Gy) WT recipient mice (CD45.1^+^CD45.2^+^). 7 weeks post‐transplantation, thymi and spleens from recipient mice were harvested for FACS analysis. A, Representative FACS plots of CD4 and CD8 staining for thymocytes and splenocytes. B, The percentage of donor CD4^+^ T cells in thymus. B, The percentage of donor CD8^+^ T cells in thymus. C, The percentage of donor CD4^+^ T cells in spleen. D, The percentage donor CD8^+^ T cells in spleen. A‐E, Results shown are from one experiment representative of three independent experiments with a total of nine mice per group. **P < .01 was considered extremely statistically significant

To confirm the intrinsic role of Med1 in CD8^+^ T cell population in the spleen, we adoptively transferred a mixture of bone marrow cells from WT (CD45.2^+^) and Med1^TKO^ mice (CD45.1^+^) at a 1:1 ratio into lethally irradiated CD45.1^+^CD45.2^+^ WT recipient mice. 7 weeks after transfer, thymocytes and splenocytes were harvested from bone marrow chimaeras. Med1‐deficient donor cells reproduced the phenotype in Med1^TKO^ mice (Figure 2A). Med1‐deficient donors exhibited similar percentages of CD4^+^ and CD8^+^ T cells in the thymus (Figure 2B,C), while there was a reduction of both CD4^+^ and CD8^+^ T cells in the spleen compared with WT donor cells (Figure 2D,E). Therefore, Med1 deletion intrinsically impairs T cell populations in the periphery.

### 3.3 Reduction of CD8^+^ T cells in spleen is due to defect in homeostasis

**FIGURE 3:**
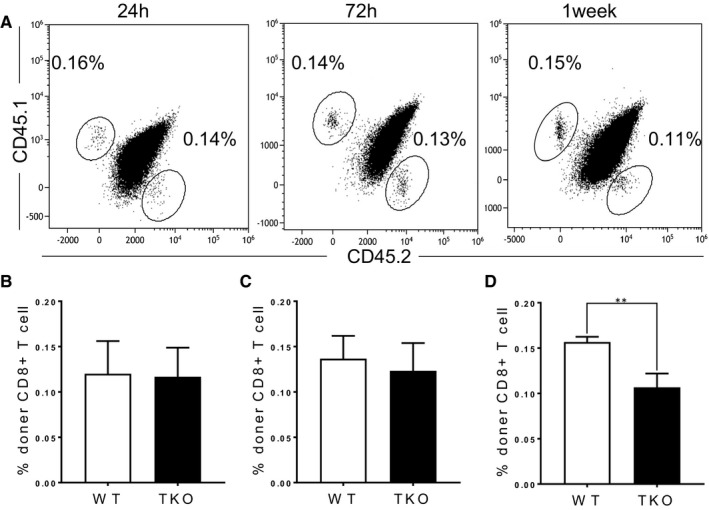
Effect of Med1 on the migration and maintenance of CD8^+^ T cells. CD8^+^ T cells from WT (CD45.1+) and Med1^TKO^ mice (CD45.2^+^) were mixed at a 1:1 ratio and transferred into sublethally irradiated WT recipient mice (CD45.1^+^CD45.2^+^). At indicated time points, spleens from recipient mice were harvested for FACS analysis. A, Representative FACS plots of CD45.1 and CD45.2 staining in CD8^+^ T cells. The percentage of donor CD8^+^ T cells in the spleen at 24 h (B), 72 h (C) and 1 week (D). A‐D, Results shown are from one experiment representative of three independent experiments with a total of nine mice per group. **P < .01 was considered extremely statistically significant

To address whether the reduction of CD8^+^ T cell population is caused by migration from the thymus or maintenance in the periphery, we co‐transferred WT (CD45.1^+^) and Med1‐deficient (CD45.2^+^) naïve CD8^+^ T cells at a 1:1 ratio into sublethally irradiated WT recipients (CD45.1^+^CD45.2^+^). Med1‐deficient CD8^+^ T cells showed the same percentage as WT cells in the spleens at 24 hours (Figure 3A,B) and 72 hours post‐transfer (Figure 3A,C). However, Med1‐deficient CD8^+^ T cells significantly decreased in the spleens one week following transfer (Figure 3A,D). Together, these findings demonstrate that the reduction of Med1‐deficient CD8^+^ T cells in periphery is due to homeostatic defect.

### 3.4 Med1 deletion enhanced apoptosis in Med1‐deficient CD8^+^ T cells

**FIGURE 4:**
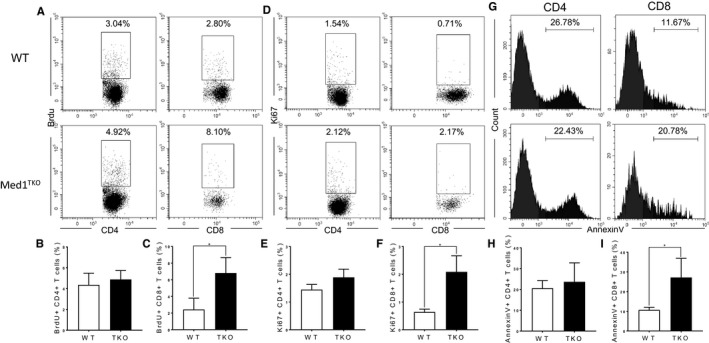
Effect of Med1 on T cell apoptosis and proliferation. 1 mg of BrdU per mouse was i.p. injected to WT and Med1^TKO^ mice. 4 h post‐injection, spleens were harvested for FACS analysis. A, Representative FACS plots of CD4, CD8 and BrdU staining in CD4^+^ and CD8^+^ splenocytes. The statistical percentage of BrdU^+^ cells in CD4^+^ (B) and CD8^+^ T cells (C). D, Representative FACS plots of CD4, CD8 and Ki67 staining in CD4^+^ and CD8^+^ splenocytes. The statistical percentage of Ki67^+^ cells in CD4^+^ (E) and CD8^+^ T cells (F). G, Representative histograms of Annexin V staining in CD4^+^ and CD8^+^ splenocytes. The statistical percentage of Annexin V^+^ cells in CD4^+^ (H) and CD8^+^ T cells (I). A‐I, Results shown are from one experiment representative of three independent experiments with a total of nine mice per group. *P < .05 was considered statistically significant

To determine the mechanism for CD8^+^ T cell reduction in the periphery, we evaluated proliferation and apoptosis using BrdU incorporation, Ki67 and Annexin V staining in peripheral T cells. Our data showed a low expression of BrdU and Ki67 in both CD4^+^ and CD8^+^ T cells from the spleens (Figure 4A,D). The percentages of BrdU^+^ and Ki67^+^ were similar in CD4^+^ T cells from WT and Med1^TKO^ group (Figure 4B,E), whereas the percentages of BrdU^+^ and Ki67^+^ cells were slightly higher in Med1‐deficient CD8^+^ T cells than WT T cells (Figure 4C,F). Importantly, we showed a significant increase of Annexin V^+^ cells in CD8^+^ T cells (Figure 4G,I), but not in CD4^+^ T cells from Med1^TKO^ in comparison with that from WT mice (Figure 4H). The data demonstrate that Med1 is required for CD8^+^ T cell survival under homeostatic conditions.

### 3.5 Loss of Med1 significantly impaired IL‐7Rα signalling and activated Bim expression

**FIGURE 5:**
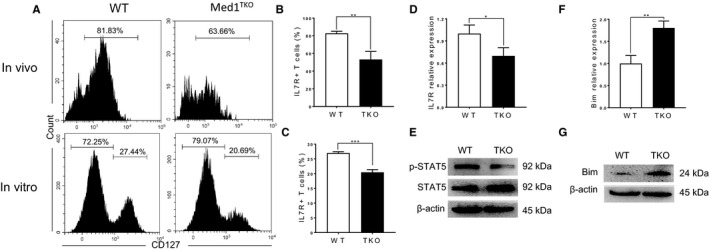
Med1 is required for IL‐7Rα expression and downstream signalling. Freshly isolated splenocytes from WT and Med1^TKO^ mice (A, top panel), or purified CD8^+^ T cells from WT and Med1^f/f^ERcre mice treated with three doses of tamoxifen (10 mg/mouse) were cultured with 10 ng/ml IL‐7 for 7 days. (A, low panel), stained with CD8, CD44, CD62L and IL‐7Rα antibodies. n = 3 mice. A, Representative histograms of IL‐7Rα staining in CD8^+^ T cells. The gates indicate CD8^+^CD44^−^CD62L^+^ cells. B, The percentage of IL‐7Rα^+^ cells in naïve CD8^+^ T cells freshly isolated spleens. C, The percentage of IL‐7Rα^+^cells in CD8^+^ T cells in vitro cultured with IL‐7Rα for 7 days. D, Relative fold change of IL‐7Rα transcription detected by qPCR method. E, The level of STAT5 and pSTAT5 in CD8^+^ T cells from WT and Med1^TKO^ mice measured by Western blotting. F, Relative fold change of Bim transcription detected by qPCR. G, Bim protein expression in CD8^+^ T cells from WT and Med1^TKO^ mice measured by Western blotting. A‐G, Results shown are from one experiment representative of three independent experiments with a total of nine mice per group. *P < .05, **P < .01 and ***P < .001 was considered statistically significant and extremely significant, respectively

In the context of T cell homeostasis in periphery, IL‐7Rα signalling is crucial for cell survival through activating STAT5 and suppressing apoptosis pathway.^6^, ^7^ To determine whether Med1 deletion affects IL‐7Rα signalling, we assessed the expression of IL‐7Rα and downstream molecules in naïve CD8^+^ T cells. Both in vivo (Figure 5A, left and B) and in vitro (Figure 5A, right and C) experiments showed that IL‐7Rα expression significantly decreased in Med1‐deficient CD8^+^ T cells. We further confirmed a decrease of IL‐7Rα at the transcription level in Med1‐deficient CD8^+^ T cells (Figure 5D). Moreover, the level of pSTAT5, the immediate downstream molecule of IL‐7Rα, was significantly decreased in Med1‐deficient CD8^+^ T cells compared with WT cells (Figure 5E). Finally, we showed that Med1‐deficient CD8^+^ T cells displayed a significant increase in *Bim* transcription (Figure 5F) and protein expression (Figure 5G) compared with WT cells (Figure 5G). Collectively, our data demonstrated that Med1 deletion impaired IL‐7Rα expression, downstream pSTAT5 level, as well as an increase of Bim expression, leading to enhanced apoptosis.

## 4 DISCUSSION

Previous studies demonstrated that naive T cell maintenance in the periphery via physiological homeostatic mechanisms primarily relies on TCR and IL‐7Rα signals. However, there is lack of detailed mechanisms underlying the maintenance of peripheral T cells. In the current study, we reported that Med1 is specifically and intrinsically required for the maintenance of CD8^+^ T cell pool in the spleen.

Med1 is essential for the development of several organs, including heart, eye, vascular and hematopoietic system.^28^, ^29^ Recently, it has been reported that Med1‐deficiency led to a reduction in CD8^+^ splenocytes in addition to a significant block in iNKT cell development without affecting thymic αβ T cells.^26^ Consistently, we observed a normal proportion of CD4^+^ and CD8^+^ T cells in the thymus, as well as a significant reduction in CD8^+^ but not CD4^+^ T cell population in spleen. These findings demonstrated that Med1 deletion intrinsically impairs the maintenance of CD8^+^ T cells in the spleen. Interestingly, the competitive chimeric bone marrow transfer experiment, Med1‐deficient CD4^+^ T cells also showed a significant reduction compared with WT T cells. This observation needs a further investigation in the future.

The maintenance of peripheral CD8^+^ T cell pool is contributed by thymocyte migration, homeostatic proliferation and survival. Our data demonstrated Med1‐deficient CD8^+^ thymocytes migrate to the spleen in similar kinetics compared with WT cells and exhibit comparable proliferation ability demonstrated by BrdU and Ki67 expression. However, Med1‐deficient CD8^+^ T cells showed a significant increase in apoptosis, contributing to reduced cellularity in the spleen. These findings are consistent with past studies demonstrating that Med1 is required for survival of other cell types and promotes tumorigenesis,^18^, ^22^, ^23^, ^24^ as well as normal embryonic development.^25^

IL‐7 binding to IL‐7Rα triggers the phosphorylation of JAK‐1 and JAK‐3,^10^ in turn activates STAT5 and promotes the expression of cell survival‐related genes, suppressing the proapoptotic proteins.^11^, ^12^, ^13^, ^14^, ^15^ Our data clearly showed that Med1 deletion diminishes IL‐7Rα expression at both the transcription and translation levels. The decrease in IL‐7Rα expression led to a reduction in STAT5 activation as well as an increase of Bim expression. This series of events contribute to an overall increased cell death in Med1‐deficient CD8^+^ T cells. However, we did not observe decreased IL‐7Rα expression in Med1‐deficient CD4^+^ T cells (Figure S1), which might explain the normal splenic CD4^+^ T cell population Med1^TKO^ mice. The basic molecular function of Med1 is to regulate gene transcription through binding of a variety of cofactors.^17^ The different effect of Med1 on CD4^+^ and CD8^+^ T cell population raises a possibility that there are differentially expressed genes responsible for the maintenance CD4^+^ and CD8^+^ T cells and these genes are bound by Med1 to activate distinct signalling pathways. These cell‐type specific mechanisms in CD4^+^ and CD8^+^ T cells require further studies.

Taken together, our studies revealed a crucial role for Med1 in the maintenance of peripheral CD8^+^ T cells. Med1 regulates CD8^+^ T cell population size by maintaining IL‐7Rα and activating STAT5 and Bim expression. Future studies should address how Med1 regulates IL‐7Rα expression.

## CONFLICT OF INTEREST

We declare no competing interests.

## AUTHOR CONTRIBUTIONS

**Lei Lei:** Data curation (lead); Writing‐original draft (lead). **Xiaofeng Yang:** Data curation (lead); Writing‐original draft (lead). **Yanhong Su:** Data curation (lead); Writing‐original draft (lead). **Huiqiang Zheng:** Methodology (equal). **Jun Liu:** Methodology (equal). **Haiyan Liu:** Methodology (equal). **Yujing Zou:** Writing‐review & editing (equal). **Anjun Jiao:** Data curation (equal). **Xin Wang:** Data curation (equal). **Cangang Zhang:** Data curation (equal). **Xingzhe Zhang:** Data curation (equal). **Jiahui Zhang:** Data curation (equal). **Dan Zhang:** Data curation (equal). **Xiaobo Zhou:** Formal analysis (equal). **Lin Shi:** Formal analysis (equal). **Enqi Liu:** Formal analysis (equal). **Liang Bai:** Project administration (lead); Writing‐review & editing (lead). **Chenming Sun:** Project administration (lead); Writing‐review & editing (lead). **Baojun Zhang:** Project administration (lead); Writing‐review & editing (lead).

## Supporting information

Fig S1

## Data Availability

All data, models and code generated or used during the study are available in the submitted article.
