# Supplementary figures and images for: Med1 controls CD8 T cell maintenance through IL‐7R‐mediated cell survival signalling
Source: J Cell Mol Med. 2021 Mar 17;25(10):4870–6. doi: 10.1111/jcmm.16465 (PMC8107092; doi:10.1111/jcmm.16465)

Fig.S1 Med1 is not required for IL-7R signaling-mediated CD4 T cells survival in vivo.

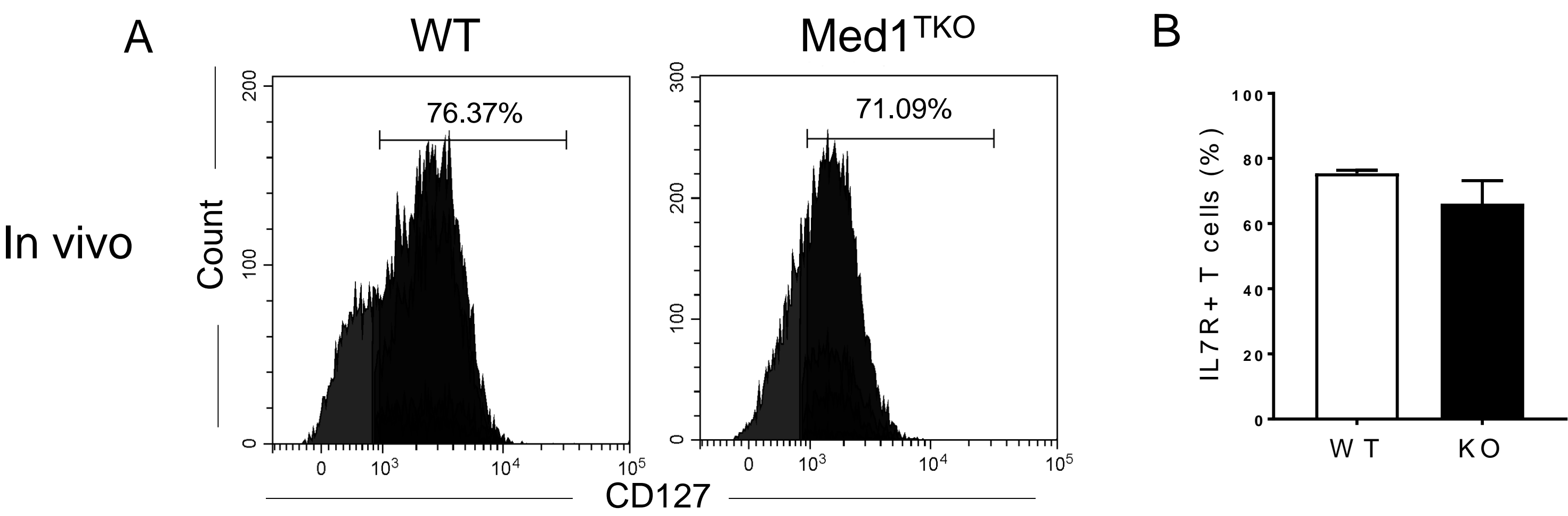

Supplement: Supplementary file 1 — Fig S1 [file JCMM-25-4870-s001.pdf]
